# The path to elimination: FEBRASGO 2023's targeted strategies against cervical cancer in Brazil

**DOI:** 10.61622/rbgo/2024EDT02

**Published:** 2024-03-15

**Affiliations:** 1 Universidade Federal de Minas Gerais Faculdade de Medicina Departamento de Ginecologia e Obstetrícia Belo Horizonte MG Brazil Departamento de Ginecologia e Obstetrícia, Faculdade de Medicina, Universidade Federal de Minas Gerais, Belo Horizonte, MG, Brazil.; 2 Faculdade de Medicina do ABC Departamento de Ginecologia e Obstetrícia Santo André SP Brazil Departamento de Ginecologia e Obstetrícia, Faculdade de Medicina do ABC, Santo André, SP, Brazil.; 3 Universidade Federal de São Paulo Escola Paulista de Medicina Departamento de Ginecologia São Paulo SP Brazil Departamento de Ginecologia, Escola Paulista de Medicina, Universidade Federal de São Paulo, São Paulo, SP, Brazil.; 4 Universidade Federal do Paraná Faculdade de Medicina Departamento de Ginecologia e Obstetrícia Curitiba PR Brazil Departamento de Ginecologia e Obstetrícia, Faculdade de Medicina, Universidade Federal do Paraná, Curitiba, PR, Brazil.; 5 Universidade Estadual de Campinas Faculdade de Medicina Departamento de Tocoginecologia Campinas SP Brazil Departamento de Tocoginecologia, Faculdade de Medicina, Universidade Estadual de Campinas, Campinas, SP, Brazil.

The battle against cervical cancer in Brazil starkly mirrors the broader global health inequity, posing significant challenges to women’s health worldwide. As the fourth most common cancer among women on a global scale, the prevalence of cervical cancer underscores the urgent need for enhanced prevention, detection, and treatment efforts. The World Health Organization (WHO) highlighted this imperative in 2018 when Director-General Dr. Tedros Adhanom Ghebreyesus issued a call to action for the elimination of cervical cancer as a public health concern—a call that was further amplified by a historic resolution by the WHO Member States in 2020, setting ambitious targets for 2030.^(1,2)^

In Brazil, the urgency of this issue is underscored by an expected annual incidence of 17,010 new cases between 2023 and 2025, ranking it as the third most common cancer among Brazilian women. The significant health burden posed by cervical cancer is unevenly distributed across the country, necessitating tailored regional responses to effectively combat the disease. The established link between cervical cancer and infection with a high-risk oncogenic type of Human Papillomavirus (HPV) further highlights the critical importance of comprehensive vaccination and screening programs.^(3-5)^

However, the challenges in the diagnosis and management of cervical cancer in Brazil are manifold. The average age of diagnosis is 48.7 years, with the majority of cases being advanced at presentation. Notably, the Southeast region reports fewer advanced cases compared to other regions, but more than half of the patients nationwide face treatment delays, contributing to a concerning early mortality rate. Despite a marginal decline in mortality rates from 2000 to 2015, the rates have since plateaued, indicating persistent obstacles in the prevention and management of the disease.^(4)^

The situation is further complicated by the shortfall in HPV vaccination coverage in Brazil, which currently falls below the WHO’s elimination goals for 2030. Although the HPV vaccine has been available for girls since 2014 and boys since 2017, vaccination coverage in Brazil has not reached the target of 90%. In 2023, coverage for girls in accumulated doses was 57.7% and for boys 27.4% considering the recommendation of a two-dose schedule in the age group 9 to 14 years.^(6)^

According data from clinical studies across multiple geographies that suggest a single-dose regimen provides significant protection against HPV, the World Health Organization (WHO) Strategic Advisory Group of Experts on Immunization (SAGE) recently recommended updating dose schedules for the HPV vaccine to 1 or 2 doses for females aged 9 to 20 years. These studies showed that single dose delivers high levels of protection similar in magnitude to multidose regimens.^(7-10)^

Moreover, while screening coverage is reportedly high, there is skepticism regarding the accuracy of these self-reported rates, and the effectiveness of current screening strategies is inconsistent across regions, with some areas still underperforming.^(5)^

Reflecting these challenges on a global scale, cervical cancer constitutes a significant proportion of female cancers. In Brazil, the mortality rate amplifies the urgency for action, with numerous factors exacerbating the disease’s progression. It is within this context that FEBRASGO’s 2023 initiative emerges, offering a robust, multidimensional strategy to meet the WHO’s targets. By integrating vaccination, screening, and treatment, FEBRASGO’s 2023 aims to significantly contribute to the global effort to eradicate cervical cancer, positioning Brazil as a vanguard in public health innovation and societal advancement.

FEBRASGO reaffirms its steadfast support for the WHO’s call to action for the eradication of cervical cancer. Following an extensive discussion forum on HPV vaccination, cervical cancer screening, and patient treatment, we advocate for an integrated approach founded on three fundamental pillars: prevention through comprehensive vaccination, early detection through effective screening, and timely, equitable treatment access. This strategy embodies our commitment to advancing healthcare for all Brazilian women, bridging the gap in global health equity, and ultimately striving for the elimination of cervical cancer as a public health problem.

## VACCINATION

### Strategies to increase vaccination coverage for women and men:

- Redefine communication about sexually transmitted infections (STI) by presenting the vaccine as protection against cervical cancer and other neoplasm locations instead against STI;
- Promote the benefits and safety of vaccination through social networks, in addition to combating fake news;
- Promote vaccination in adult individuals;
- Actively search for individuals eligible for vaccination and incentive for physicians to get orientation and prescription in the offices;
- Considering the specificities of the priority age group for the HPV vaccine, foster the availability of hours and service offerings in vaccination rooms on weekends.

### School vaccination:

- Support, strengthen, and expand vaccination programs in schools; considered essential.

### Vaccination scheme:

- Expand the vaccination scheme from 2 doses (interval of 0 and 6 months) to individuals aged 9 to 20 years;
- Consider the administration of a single dose in boys and girls according to OMS orientation and then to achieve the vaccination coverage suggested by the WHO.

### Specific situations:

- Disseminate information to HIV people’s NGOs about the importance of vaccination; expand the dissemination of vaccination criteria for women and men up to 45 years old living with HIV, cancer patients, and transplant recipients, along with the health services that provide care to these groups and civil entities (NGOs, support foundations, etc.);
- Recommend the HPV vaccine as soon as possible for women with high-grade lesions on the cervix diagnosed histologically and before or after surgery (LLETZ, LEEP or conization) when indicated.^(11)^

### Vaccination training:

- Develop vaccination training and counseling strategies directed at doctors (gynecologists, family and community doctors, pediatricians), other health professionals, and civil society;
- Encourage the expansion of knowledge about vaccination to teachers and education professionals with strategies and training to guide parents and students;

### FEBRASGO Advocacy:

- Encourage integration between the Ministries of Education and Health for the dissemination of information and the development of vaccination strategies;
- Expand and qualify the actions of the Health in Schools Program (PSE) specifically regarding the concepts of primary and secondary prevention of cervical cancer.

## SCREENING

Encourage the expansion of coverage and organization of the cervical cancer screening program and the entire care line:

- Essential to have a unique population-based registration system, integrated with e-SUS, accessible at all stages of the official screening program: collection registration, result consultation, developments/follow-up, and management;
- Increase the coverage and improve the organization of screening with periodic analyzing programs in Brazil;
- Train health professionals (community health agents, doctors, and nurses) for appropriate referral after altered test results;
- Expand access and standardize care protocols;
- Actively search for women not screened;
- Reinforce the target age range of the population screening from 25-64 years.

### Screening test:

- Support the change from primary screening test to DNA-HPV test and with evaluation of the best protocol to adaptation to using in Brazil;
- Consider the screening test in the context of the entire cervical cancer control program, including actions regarding screening, secondary and tertiary care. Organize and record both the patients and the processes.
- Encourage self-collection of vaginal samples for molecular tests to increase coverage rates among groups with limited access to health services and women resistant to traditional gynecological examination;
- Educate women and health professionals about the meaning of a positive HPV test and the necessary developments for complementary investigation;
- Consider regional differences with the possibility of distinct models for specific realities.

### Access in remote areas:

- In remote locations, expand the discussion for the implementation of strategies based on the "see and treat" approach, considering the use of new technologies with rapid tests (point of care), visual inspection with acetic acid (VIA), digital colposcopy devices with remote reports, considering treatments with ablative or excisional techniques.

## TREATMENT

### Training in Diagnosis and Treatment:

- Train gynecologists to diagnose and treat pre-invasive lesions of the cervix;
- Consider "see-and-treat" in specific situations and ages;
- Create the field of practice in Gynecologic Oncology;

### Support to patient groups:

- Work alongside groups of patients with cervical cancer;

### Access in remote areas:

- In hard-to-reach locations, promote training for the application of VIA and "see and treat" methods, including ablative (as cryotherapy and others techniques) and excisional techniques.

### Assistance to women with cervical cancer:

- Establish more reference centers in Pathology of the lower genital tract (PTGI) and Gynecologic Oncology;
- Create means to welcome and assist women with cervical cancer throughout their journey, from diagnosis to palliative care.

### General Premises:

- Always consider diversity, equity, and inclusion in all pillars for the control of cervical cancer;
- Mobilize civil society, the press, and media about the burden of cervical cancer in Brazil.

### Database and metrics:

- Improve records and indicators of cervical cancer in Brazil;
- Audit and periodically review protocols, strategies, actions, and results;
- Encourage epidemiological research, innovation, and technology related to cervical cancer in Brazil.

### Advocacy:

- Reinforce FEBRASGO’s participation in advocacy for cervical cancer in Brazil.

As we move towards the WHO’s 2030 milestone for cervical cancer elimination, the comprehensive actions proposed offer a robust framework for Brazil’s national strategy. By intertwining advanced vaccination campaigns, widespread and accessible screening, and equitable treatment options, the plan addresses the multifaceted nature of cervical cancer prevention and care. These concerted efforts are tailored to meet the diverse needs across Brazil’s socio-cultural spectrum, aiming to significantly reduce both the incidence and mortality rates of this preventable disease. This is not just a health initiative; it represents a collective commitment to the well-being of Brazilian women, affirming their right to a life unmarred by the threat of cervical cancer. The journey ahead relies on the steadfast implementation of these strategies and the continuous adaptation to technological and medical advancements. By adhering to the outlined actions, Brazil is on course to not only meet international targets but also to establish itself as a global exemplar in the fight against cervical cancer. This will involve an alliance of stakeholders across all levels of society, each contributing to a narrative of proactive change and determination. With these efforts, the eradication of cervical cancer from the public health agenda in Brazil is an attainable reality, marking a significant step towards bridging the global health equity divide and securing a healthier future for all Brazilian women.

## References

[1] World Health Organization (WHO) (2023). Cervical cancer elimination initiative: from call to action to global movement.

[2] World Health Organization (WHO) (2020). Global strategy to accelerate the elimination of cervical cancer as a public health problem.

[3] Ministério da Saúde (2022). Instituto Nacional de Câncer (INCA). Estimativa 2023: incidência de câncer no Brasil.

[4] Paulino E, Melo AC, Silva AL, Maciel LF, Thuler LC, Goss P (2020). Panorama of gynecologic cancer in Brazil. JCO Glob Oncol.

[5] Corrêa FM, Migowski A, Almeida LM, Soares MA (2022). Cervical cancer screening, treatment and prophylaxis in Brazil: current and future perspectives for cervical cancer elimination. Front Med.

[6] Ministério da Saúde (2023). Campanha Nacional de Multivacinação em crianças e adolescente. Sistema de Informação do Programa Nacional de Imunização: dados acumulados desde a implantação da vacina até 2022.

[7] Barnabas RV, Brown ER, Onono MA, Nojorose B, Bukusi EA, Winer RL (2022). Efficacy of single-dose HPV vaccination among young African. NEJM Evid.

[8] Watson-Jones D, Changalucha J, Whitworth H, Pinto LA, Mutani P, Indangasi J (2022). Immunogenicity and safety results comparing single dose human papillomavirus vaccine with two or three doses in Tanzanian girls - the DoRIS randomised trial [Preprint].

[9] Basu P, Malvi SG, Joshi S, Bhatla N, Muwonge R, Lucas E (2021). Vaccine efficacy against persistent human papillomavirus (HPV) 16/18 infection at 10 years after one, two, and three doses of quadrivalent HPV vaccine in girls in India: a multicentre, prospective, cohort study. Lancet Oncol.

[10] Porras C, Schiller JT, Kemp T, Herrero R, Wagner S, Boland J (2020). Evaluation of durability of a single dose of the bivalent HPV vaccine: The CVT Trial. J Natl Cancer Inst.

[11] Di Donato V, Caruso G, Petrillo M, Kontopantelis E, Palaia I, Perniola G (2021). Adjuvant HPV vaccination to prevent recurrent cervical dysplasia after surgical treatment: a meta-analysis. Vaccines (Basel).

